# Purine Metabolism Dysfunctions: Experimental Methods of Detection and Diagnostic Potential

**DOI:** 10.3390/ijms24087027

**Published:** 2023-04-10

**Authors:** Arrigo F. G. Cicero, Federica Fogacci, Valentina Di Micoli, Cristina Angeloni, Marina Giovannini, Claudio Borghi

**Affiliations:** 1 Cardiovascular Internal Medicine Unit, IRCCS Azienda Ospedaliero Universitaria di Bologna, 40138 Bologna, Italy; 2 Hypertension and Cardiovascular Risk Research Group, Medical and Surgical Sciences Department, Alma Mater Studiorum University of Bologna, 40138 Bologna, Italymarina.giovannini3@unibo.it (M.G.); 3 Medical and Surgical Sciences Department, Alma Mater Studiorum University of Bologna, 40138 Bologna, Italy; 4 Department for Life Quality Studies, Alma Mater Studiorum University of Bologna, 47921 Rimini, Italy; cristina.angeloni@unibo.it

**Keywords:** uric acid, allantoin, xanthine, purine metabolism

## Abstract

Purines, such as adenine and guanine, perform several important functions in the cell. They are found in nucleic acids; are structural components of some coenzymes, including NADH and coenzyme A; and have a crucial role in the modulation of energy metabolism and signal transduction. Moreover, purines have been shown to play an important role in the physiology of platelets, muscles, and neurotransmission. All cells require a balanced number of purines for growth, proliferation, and survival. Under physiological conditions, enzymes involved in purines metabolism maintain a balanced ratio between their synthesis and degradation in the cell. In humans, the final product of purine catabolism is uric acid, while most other mammals possess the enzyme uricase that converts uric acid to allantoin, which can be easily eliminated with urine. During the last decades, hyperuricemia has been associated with a number of human extra-articular diseases (in particular, the cardiovascular ones) and their clinical severity. In this review, we go through the methods of investigation of purine metabolism dysfunctions, looking at the functionality of xanthine oxidoreductase and the formation of catabolites in urine and saliva. Finally, we discuss how these molecules can be used as markers of oxidative stress.

## 1. Introduction

Uric acid (UA) is the final product of both the exogenous pool of purines and their endogenous metabolism. The exogenous pool varies significantly with diet, to which animal protein significantly contributes. Endogenous UA production, instead, mainly comes from the liver, intestine, and other tissues, such as muscle, kidney, and vascular endothelium [1].

UA is a heterocyclic organic compound with the formula C_5_H_4_N_4_O_3_ (7,9-dihydro-1*H*-purine-2,6,8(3*H*)-trione) and s molecular weight of 168 Daltons, and it is the final metabolic product of purine metabolism in humans.

**Figure 1:**
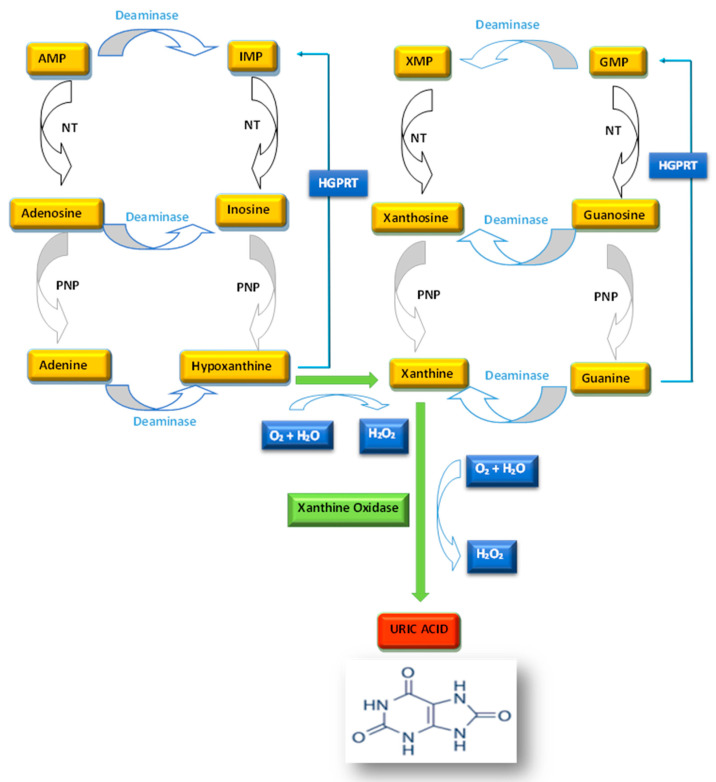
Biosynthesis of uric acid. AMP, adenosine monophosphate; GMP, guanine monophosphate; HGPRT, hypoxanthine-guanine phosphoribosyltransferase; IMP, inosine monophosphate; NT, 5′-nucleotidase; PNP, purine nucleoside phosphorylase; XMP, xanthosine monophosphate.

Many different enzymes are involved in the conversion of the nucleobases adenine and guanine to UA. Adenosine monophosphate (AMP) can be converted to inosine by two different mechanisms: (i) AMP deamination, followed by de-phosphorylation; or (ii) dephosphorylation, followed by deamination. Guanine monophosphate (GMP) is converted to guanosine by a nucleotidase. The nucleosides inosine and guanosine are further converted to the purine bases hypoxanthine and guanine, respectively, by purine nucleoside phosphorylase (PNP). Hypoxanthine is then oxidized to form xanthine by xanthine oxidase (XO), and guanine is deaminated to form xanthine by guanine deaminase. Xanthine is oxidized again by XO to form UA, being the end degradation product of purine nucleotides [2] (Figure 1).

**Figure 2:**
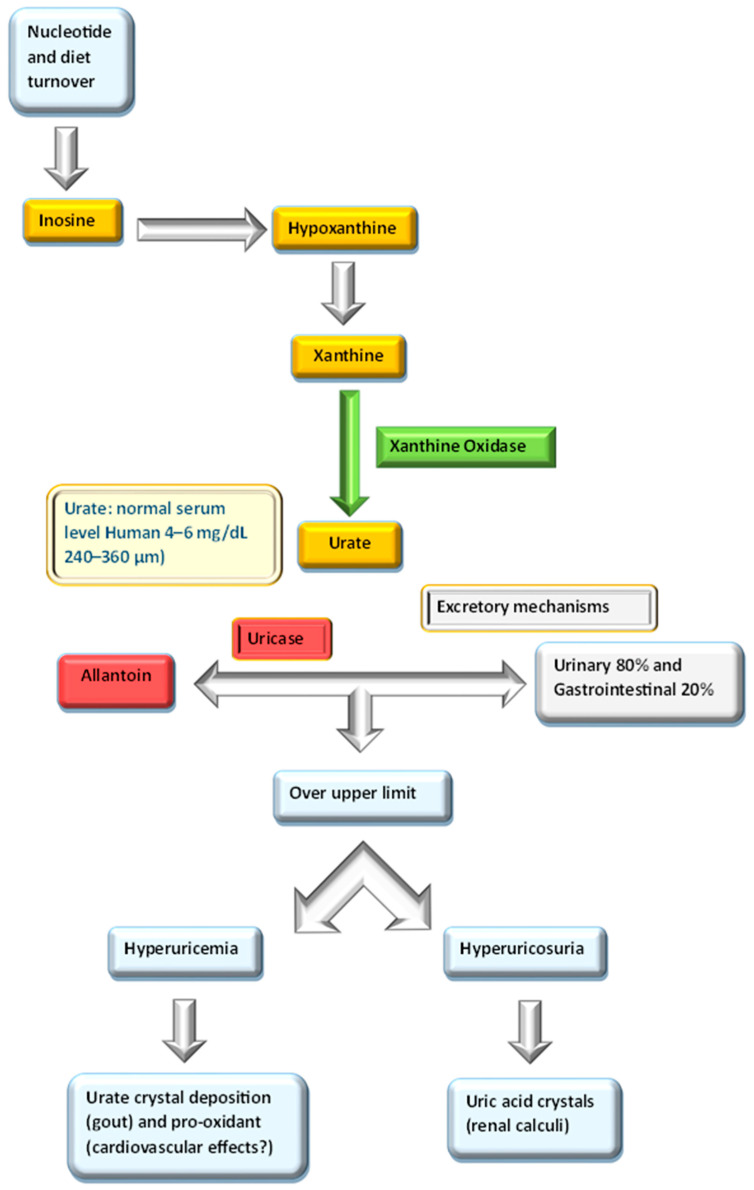
Pathways of urate homeostasis.

The pathway of urate homeostasis is resumed in Figure 2. Physiologically, most of the daily disposal of UA occurs through the kidneys. In most mammals, the enzyme uricase (urate oxidase) further oxidizes UA into allantoin. However, during the early stages of hominid evolution, the uricase gene probably underwent a functional mutation, with the consequence that humans cannot oxidize UA into the more soluble compound allantoin because of the lack of the uricase [3].

The deposition of microcrystals of monosodium urate monohydrate or UA in several parts of the body leads to the development of gastroenteritis and gout [4]. Gout, in particular, may result in one or a combination of acute arthritis (a gout flare), chronic arthritis (chronic gouty arthritis), and tophi (tophaceous gout) [5]

UA concentration can be measured in serum, plasma, urine, and exhaled breath condensate. The laboratory methods used to determine the serum UA concentration are deeply described in the next section.

Several epidemiological studies have shown that a correlation exists between elevated levels of serum UA (SUA), hypertension, and chronic kidney disease (CKD) [6,7]. A longitudinal cohort population study showed that baseline SUA levels greater than 6.5 mg/dL are associated with a 25% increased risk of hypertension, while meta-analysis pooling data from 55,607 individuals showed a 13% increase in hypertensive events for every 1 mg/dL increase in SUA levels [8]. The most recent observations from the large Uric acid Right for heArt Health (URRAH) project have reinforced that the actual hyperuricemia cutoff (>6 mg/dL in women and 7 mg/dL in men) is principally based on the saturation point of UA, though the negative impact on cardiovascular system could occur also at lower levels [9,10]. However, according to the latest findings from the URRAH study, the SUA/serum creatinine (sCr) ratio could be more appropriate than SUA alone in predicting the individual cardiovascular risk, as it includes the weight of renal function, which is intimately linked to SUA [11]. In particular, the SUA/sCr cutoff of >5.35 in the frame of a nationwide study can stratify individuals destined to experience incident cardiovascular events during a long-lasting follow-up from those destined to be events-free. Remarkably, this cutoff is a dynamic value with the tendency to increase with increasing SUA/sCr, being a low-cost reliable tool that might be used systematically in epidemiological setting, as well as in clinical practice [11].

In light of the current evidence, the multifactorial mechanism linking hyperuricemia to cardiovascular and renal diseases involves both SUA level and XO activity [12], with the XO inhibitors (XOIs) being the most potent available UA-lowering drugs, with documented beneficial effects on both cardiovascular and renal systems [13]. The benefits depending on treatment with XOIs may rely on XOIs’ antioxidant properties other than SUA reduction, as the XOIs secondarily inhibit the production of reactive oxygen species by the XO [14]. Population-based cohort studies identified reductions in all-cause and heart-failure-related mortality following treatment with allopurinol [15]. In general, several observational studies suggest that allopurinol use is associated with lower mortality, but association does not prove causality [16].

## 2. Hyperuricemia and Hypouricemia

UA is the product of purine metabolism in humans and has antioxidant properties that can be both protective and pro-oxidant, depending on its chemical microenvironment [17]. Hyperuricemia predisposes patients to gout through the formation of urate crystals, but it has also been linked to hypertension, atherosclerosis, insulin resistance, and diabetes, independently from crystal formation [18]. In effect, the oxidative stress induced by high SUA levels through the inhibition of the endothelial nitric oxide (NO) bioavailability is supposed to result in reduced endothelium-mediated vasodilatation and lead to decreased blood flow in insulin target tissues [19]. Consequently, insulin sensitivity in the skeletal muscle is lowered, and hyperinsulinemia and insulin resistance occur in the long term. In the adipose tissue, UA induces the expression of pro-inflammatory cytokines and negatively modulates activity of the insulin sensitizer PPAR-γ (peroxisome proliferator-activated receptor gamma) [20]. Finally, at systemic levels, the induced endothelial dysfunction results in increased peripheral arterial resistance, which also contributes to smooth muscle cell proliferation in the media [21].

Today, hyperuricemia is a common finding in individuals with metabolic syndrome [22], being also associated with visceral adiposity [23,24]. In this context, there is also a need to recognize that there is an inverse correlation between insulin resistance and decreased renal clearance of UA, which, in turn, is associated with elevated uricemia [25].

In humans, uricase inactivation causes the occurrence of much higher urate levels (≈240–360 μM) than other mammals (e.g., ≈30–50 μM in mice). It has been proposed that higher serum levels of urates may be a selective advantage in hominid evolution because of its antioxidant effects [22].

At the same time, however, there are situations that can cause hypouricemia, such as hereditary xanthinuria, which is caused by a deficiency in the enzyme xanthine dehydrogenase/oxidase and finally, resulting in reduced UA production [26]. This autosomal recessive disease is characterized by hypouricemia, low urinary urate concentrations, and high urinary xanthine concentrations, which lead to the development of xanthine kidney stones [27].

Drugs that lower urate levels by inhibiting XO (e.g., allopurinol and febuxostat) can also cause hypouricemia [28]. A number of genetic mutations can also cause renal hypouricemia, such as mutations in SLC22A12, which encodes URAT1, and SLC2A9, which encodes GLUT9 [29,30].

## 3. Uric Acid as Antioxidant

Historically, UA has always been considered an inert waste product that, when highly concentrated, crystallizes, forming kidney stones and causing gouty arthritis [27]. Recently, it has been more properly recognized as a potent antioxidant that eliminates oxygen radicals, singlet oxygen, and peroxynitrite and chelates transition metals, reducing, for example, iron ion-mediated oxidation of ascorbic acid. Thus, urate accounts for about half of the antioxidant capacity of human plasma, and its antioxidant properties are as potent as those of ascorbic acid [31,32].

UA is presumed to act as a potent antioxidant in the extracellular environment, having also pro-oxidant properties inside the cell, where it stimulates nicotinamide adenine dinucleotide phosphate (NADPH) oxidase [33]. Most recently, UA has been reported to induce endothelial dysfunction by increasing oxidative stress and decreasing the bioavailability of endothelial NO [34]. Therefore, UA reduction with allopurinol may improve endothelial dysfunction in humans [35].

Consistent with the abovementioned studies, hypouricemia has been associated with inflammatory and degenerative diseases, such as acute organ transplantation disease, Huntington’s disease, Alzheimer’s disease, Parkinson’s disease, and multiple sclerosis, attributing these associations to a reduced antioxidative capacity [36,37].

About two-thirds of all UA in humans is excreted in urine and one-third in the gastrointestinal tract [38]. Urate transporter 1, which is located on the luminal side of proximal tube cells (URAT1; SLC22A12), reabsorbs filtered UA at the glomerular level [39], while glucose transporter 9 (GLUT9; SLC2A9), through the basal side of cells, allows intracellular UA to exit (Table 1) [36,40]; otherwise, UA secretion may be mediated by ATP-binding cassette transporter subfamily G member 2 (ABCG2) and sodium-dependent phosphate transporter 1 (NPT1; SLC17A1) and 4 (NPT4; SLC17A3) [41,42]. Interestingly, studies on polymorphisms of these transporters suggest that renal overload hyperuricemia is a novel pathophysiological mechanism in gout [43,44].

The ABCG2 transport protein is encoded by the ABCG2 gene (chr4: 88090264-88231417c) and is a member of the ATP-binding cassette (ABC) transporter superfamily, in which substrate transfer is coupled to ATP hydrolysis. In addition to its role in urate transport, the ABCG2 protein has also been characterized as a xenobiotic transporter and a resistance factor to chemotherapeutics [45].

In humans, variants of the protein with reduced transport capacity are associated with increased renal excretion of urate. The ABCG2 messenger RNA is expressed in different tissues, such as in the intestine, brain, and placenta. Brain-level expression is suggestive of the demonstrated association between uricemia levels and susceptibility to certain neurodegenerative diseases [46,47], while ABCG2 expression in the placenta might play a role in the metabolism of urate produced by the fetus to protect it from harmful substances [48].

Thus, the identification of genetic variants could help in the prevention and intervention of some diseases. To date, a number of genome-wide association studies (GWASs) have been conducted to discover loci related to hyperuricemia and gout, mostly related to UA transporter proteins such as the widely studied URAT1 and GLUT9, and some of them have been used as targets for drug development. Attention should also be paid to the interconnection between hyperuricemia/gout and other diseases, such as metabolic syndrome and cardiovascular disease, as well as the role of genetic factors in these diseases. Elucidation of these genetic aspects will contribute to improved clinical outcomes and precision medicine [49].

## 4. The Key Role of Xanthine Oxidase as a Biomarker of Metabolic Disorders

The inadequate activation of XO can increase reactive oxygen species, resulting in oxidative-stress-induced injury in different tissues because, in mammals, xanthine oxidoreductase (XOR) can be reversibly converted into two different forms, XDH and XO. XDH reduces NAD+ to NADH, whereas XO produces hydrogen peroxide and superoxide [50]. XOR is expressed as the XDH form in different tissues by escaping into the blood through different stimuli converting into the XO form. XO released from an organ into plasma is partially bound to sulfated glycosaminoglycans on the surface of vascular endothelial cells and can act as surveillance for foreign pathogens under physiological conditions [51]. Excessive activation of endothelium-bound XOR can produce peroxynitrite from nitric oxide and impair the vasodilatory reaction. Recently, a sensitive and accurate assay for plasma XOR activity in humans was established using stable isotopes, liquid chromatography, and triple quadrupole mass spectrometry [52].

Thus, it has been shown that plasma XOR activity is independently associated not only with hyperuricemia but also with obesity, hepatic dysfunction, smoking, dyslipidemia, and insulin resistance in the general population, suggesting that plasma XOR activity could be a new metabolic biomarker [53]. Plasma XOR activity has also been shown to be independently associated with several adipokines, including adiponectin, fibroblast growth factor 21 (FGF21), and fatty-acid-binding protein 4 (FABP4) in the general population [54,55]. In addition, an annual change in plasma XOR activity has been reported to be independently associated with changes in liver enzymes and body weight [56].

One of the critical problems with measurements in purine metabolism is that the time before plasma separation can affect the concentrations of xanthine and hypoxanthine via leakage from erythrocytes. A previous study using blood samples incubated with ethylenediamine tetraacetic acid dipotassium (EDTA-2K) showed that waiting 3 h before plasma separation did not cause a significant increase in hypoxanthine or xanthine levels. Notably, it has been reported that a commercially available tube (PAXgene Blood DNA tube; Becton, Dickinson and Company, Franklin Lakes, NJ, USA) for blood sampling allows for accurate hypoxanthine and xanthine measurements regardless of the time elapsed since plasma separation [57,58].

In the study by Furuhashi M. et al., hypoxanthine concentration—though not xanthine concentration—was independently associated with body mass index and smoking habit, using plasma samples collected in EDTA-2K-coated tubes and centrifuged within 3 h and after adjustment for age, sex, and use of anti-hyperuricemic drugs in the general population [59]. The independent association of hypoxanthine with obesity suggests that a major source of hypoxanthine is adipose tissue in obesity. It has been reported that nicotine degrades the apurin-rescuing enzyme, hypoxanthine-guanine phosphoribosyltransferase (HGPRT), and that smoking lowers the activity of HGPRT, suggesting an increase in plasma levels of hypoxanthine by inhibition of the rescue pathway [60]. The xanthine level has also been shown to be independently associated with alanine aminotransferase as a liver enzyme, hypoxanthine, and plasma XOR activity [60]. These results suggest that the main source of xanthine and XOR is the liver [60].

## 5. Methods of Uric Acid Detection

Given the large epidemiological and clinical relevance of UA levels, developing quick and accurate UA detection methods would be important. SUA dosage has a relatively large pre-analytical variability due to biorhythm (especially in adult men) [61], freezing [62], and eventual hemolysis [63]. The recently developed detection methods for measuring the UA level in human serum and urine samples, saliva, nails, and tears include electrochemical methods (voltammetry, electrochemiluminescence, and surface plasmon resonance), spectral (ultraviolet absorption and fluorescence), chromatographic methods (liquid and gas phase), capillary electrophoresis and isotope dilution (mass spectrometry, etc.), and other methods [64,65,66]. The enzyme-based strategy is a highly interference-resistant and high-sensitivity method, but the detection steps are complex. Non-enzymatic strategies seem to have better development potential because of their simple, direct, rapid, and effective response. Researchers also have to overcome several problems, such as weak anti-interference and impracticality. In short, all methods have their advantages and disadvantages, depending on the needs of the experiment. In recent years, researchers have focused on electrochemical detection for UA concentration by means of color changes.

These scientific research results could prove to be a breakthrough in the field of test strips or test kits because of its rapid response, high sensitivity, and simplicity of operation [67].

## 6. Detection of Uric Acid in Urine

The quantitative analysis of relevant metabolites in biofluids such as urine is often a tedious procedure, as it usually requires extraction, purification, or preconcentration. For example, solid-phase extraction is often required for the analysis of methylxanthines in urine. Moreover, prior to urate determination in urine, urine alkalinization is required because urate crystallizes at a pH below 5.5 [68].

In the work of Rodriguez at al., a rapid and highly sensitive “dilute and shoot” method combining ultra-high-performance liquid chromatography and high-resolution mass spectrometry (UHPLC/HRMS) was validated for the urinary determination of twelve analytes: uric acid, hypoxanthine, xanthine, 1-methyluric acid, 1,3-dimethyluric acid, 1-methylxanthine, 3-methylxanthine, 7-methylxanthine, theophylline, theobromine, paraxanthine, and caffeine [69]. These analytes are the main physiological metabolites of caffeine, theobromine or theophylline, or the end products of purine catabolism [65,70].

Recently, a new quantitative assay was developed to simultaneously measure purine metabolites and creatinine in biobanked urine by liquid chromatography–tandem mass spectrometry (LC-MS/MS). The newly developed LC-MS/MS method allows for a reliable quantitative assessment of xanthine, hypoxanthine, allantoin, UA, and creatinine, with intra-day and inter-day coefficients of small variation for all the analytes, not exceeding 1% and 10%, respectively The proposed pre-analytical processing makes the method suitable for both fresh and biobanked urine stored frozen at −80 °C for at least 5.5 years [71].

## 7. Uric Acid Leaching in Saliva

Saliva is a very promising diagnostic matrix because its noninvasive sampling is characterized by cost-effectiveness, reduced complexity of analysis, and lower risk of patient infection, which are all clear advantages.

The UA level in the saliva of patients with oral diseases is considered a biomarker of antioxidant defense against oxidative stress, so that monitoring salivary UA levels is important for both diagnostic purposes and treatment programs [72]. Some researchers also report a linear correlation between UA levels in blood serum and saliva [73].

The paper by Bukharinova MA et al. describes the development of a carbon veil electrode (CVE) for the determination of UA in saliva [74]. The results support the use of the sensor for the rapid determination of UA in artificial and human saliva. The RSD does not exceed 3.9%, and the recovery is 96–105% [74]. UA contributes significantly to the antioxidant activity (AOA) of saliva (≈60%). In addition to the high analytical characteristics, the important advantages of the proposed CVE act are the simple, scalable, and inexpensive production technology and the absence of additional complex and time-consuming editing operations [73].

The evaluation of salivary UA levels can contribute to the noninvasive diagnosis of a number of serious diseases. Increased salivary UA concentration is associated with cancer, infection by human immunodeficiency virus (HIV), gout, and hypertension. In contrast, low UA levels are associated with Alzheimer disease, the progression of multiple sclerosis, and mild cognitive impairment [75].

Additionally, Liu XY et al. recently described an HPLC-MS/MS method as a fast, sensitive, and accurate tool to simultaneously determine UA and creatinine in human saliva [76]. Other techniques have been used for the detection of UA in saliva, such as liquid chromatography with electrochemical detection [77], capillary electrophoresis with electrochemical detection, miniaturized capillary electrophoresis with amperometric detection, and the fluorometric determination [78,79,80].

## 8. Evaluation of Xanthine Oxidoreductase (XOR) Activity

Studies of pathological mechanisms and characterization of XOR inhibitors, such as allopurinol, febuxostat, and topiroxostat, require accurate and sensitive measurements of XOR activity. However, established assays have some disadvantages, such as susceptibility to endogenous substances such as UA, xanthine, or hypoxanthine.

Murase T. et al. developed a new XOR activity assay by using a combination of high-performance liquid chromatography (LC) and high-resolution mass spectrometry (HRMS) for tissues, such as liver, kidney, and plasma [81]. The stable isotope [^15^N_2_]-xanthine was used as a substrate, and the production of [^15^N_2_]-UA was determined. The production of [^15^N_2_]-UA by XOR depended on the amounts of [^15^N_2_]-xanthine and enzyme and the reaction time. Since high concentrations of endogenous xanthine and hypoxanthine affect the activities of XOR, a multicomponent analysis by LC/HRMS was employed to improve the accuracy of the XOR activity assay. Subsequently, the quantification of [^15^N_2_]-UA was validated and showed good linearity, accuracy, and precision [73]. The XOR activity of ICR mice was measured using [^15^N_2_]-xanthin and LC/MS, and it was found to be 38.1 ± 0.7, 158 ± 5, and 928 ± 25 pmol/min/mg protein in plasma, kidney, and liver, respectively (mean ± standard deviation, n = 5). XOR activities were measured in the same samples by using LC/ultraviolet and LC/fluorescence (FL) methods. The level of oxidation of [^15^N_2_]-xanthine by XOR was found to be equal to that of xanthine oxidation and about 7.9–8.9 times higher than that of pterin oxidation. A good correlation was found between the activities of XOR examined by LC/MS assay with [^15^N_2_]-xanthine and those examined by LC/FL assay with pterin. Then this result suggested that, although both the LC/MS assay with [^15^N_2_]-xanthine and the LC/FL assay with pterin are useful, the former provides information on XOR activities that more directly reflects the physiological condition than the latter.

In the study by Murase T. et al., the amount of [^13^C_2_,^15^N_2_]-UA produced by XOR was determined by LC/TQMS, after synthesizing [^13^C_2_,^15^N_2_]-xanthine as the substrate, [^13^C_2_,^15^N_2_]-UA as the analytical standard, and [^13^C_3_,^15^N_3_]-UA as the internal standard [82]. The calibration curve of [^13^C_2_,^15^N_2_]-UA that was obtained by LC/TQMS in the selected reaction monitoring mode was evaluated, and the results indicated good linearity (R^2^ = 0.998, with weighting of 1/x^2^) in the range of 20 to 4000 nM. As a model reaction of less active samples, the XOR activity of serially diluted mouse plasma was measured. The XOR activity of 1024-fold diluted mouse plasma was 4.49 ± 0.44 pmol/100 μL/h (mean ± standard deviation); that is a value comparable to that expected for the XOR activity of healthy human plasma.

According to this cumulative evidence, this combined method can be used to obtain high-sensitivity measurements needed for the analysis of XOR activity on different organs or human plasma. In particular, in the study of Kurajoh M et al., plasma XOR activity was found to be positively associated with the SUA level independently of other known confounding factors affecting this level, including sex differences, estimated glomerular filtration rate (eGFR), adiponectin level, visceral fat area (VFA), and homeostatic model assessment of insulin resistance (HOMA-IR) [83].

## 9. Possible Uses of Purine Metabolism Markers in the Assessment of Oxidative Stress

Uric acid, allantoin, and oxidative stress: Experimental and clinical evidence currently shows that UA is a potential marker of oxidative stress. In particular, UA could have a potential therapeutic role as an antioxidant, being able to react with biologically relevant oxidants, such as the hydroxyl radical and hypochlorous acid (HOCl), and lead to the production of allantoin, which has been proposed to be a marker of oxidative stress itself [84,85].

In the study of Kanďár R. et al., using HPLC on plasma of erythrocytes from individuals with chronic renal failure before hemodialysis and in healthy blood donors, significantly higher levels of allantoin were found in both plasma and erythrocytes from patients with chronic renal failure, indicating that allantoin can be used as a good marker of oxidative stress [86,87]. Similarly, in the study by Zitnanová et al., patients with Down syndrome were found to have significantly higher UA levels and significantly lower levels of hypoxanthine and xanthine compared to controls [88]. This latter result suggests an increased conversion of hypoxanthine and xanthine to UA, with subsequent free radical-dependent oxidation of UA to allantoin; both mechanisms are enhanced by oxidative stress in individuals with Down syndrome. Moreover, Kanďár R. et al. monitored allantoin, uric acid, and malondialdehyde in plasma and erythrocytes immediately after strenuous exercise, showing that, after exercise plasma levels of allantoin increase by an average of 200% from baseline, plasma levels of UA more slowly increase by an average of 20%, while no significant changes in plasma malondialdehyde are detected [89]. The cumulative evidence suggests that UA is probably oxidized by reactive oxygen species into allantoin, which may be a suitable candidate as a marker of acute oxidative stress.

## 10. Main Remarks and Conclusions

Purines perform important functions in the cell, including the formation of the monomeric precursors of deoxyribonucleic acid (DNA) and ribonucleic acid (RNA). Purines are structural components of some coenzymes and modulate energy metabolism and signal transduction [90]. In addition, they have been shown to play important roles in the physiology of muscles, platelets, and neurotransmission [91]. So, under physiological conditions, the activity of enzymes involved in purine metabolism is strictly regulated and maintains a balanced ratio between their synthesis and degradation in the cell [92]. In humans, the final compound of purine catabolism is UA. In contrast, most other mammals possess the enzyme uricase, which converts UA to allantoin, which is easily eliminated through urine [92].

During the last decades, hyperuricemia has been associated with a number of human diseases, beyond gout. In fact, even though SUA has an antioxidant action in the extracellular environment, asymptomatic hyperuricemia has been associated with the development of atherosclerosis-related cardiovascular diseases, cardiac arrhythmias, heart failure, metabolic syndrome and type 2 diabetes, cognitive decline, and chronic kidney disease [93]. In cardiovascular disorders, SUA levels are also associated with the clinical severity of the diseases. Cumulative evidence from the URRAH project (as well as from other huge epidemiological studies) supports the role of SUA as an independent predictor not only of all-cause and cardiovascular mortality, but also of myocardial infarction, stroke, and heart failure [11,94].

The increased amount of UA measured in serum, saliva, and urine can be considered a negative prognostic factor for the development of acute renal failure, as well. However, despite the large number of studies on this topic, an important open question regards the identification of a disease-specific UA cutoff value.

Recent studies have shown that allantoin quantification could also be a reliable marker of oxidative stress since, like XO, it has a key role in the transformation of xanthine into UA. However, further research in humans is needed before predictive models using allantoin as a biomarker of dysfunctional purine metabolism can be developed and used in clinical practice. In fact, there are yet little data which would clearly determine which biological material and which analytical method of analyzing allantoin concentration may provide the most reliable results [95], even considering the relatively large pre-analytical variability of SUA.

Therefore, given the huge epidemiological (and, probably, pathophysiological) relevance of uric acid in determining human diseases, new more specific biomarkers of purine metabolism should be discovered in order to more correctly identify those subjects exposed to an increased disease risk.

## Figures and Tables

**Table 1:** Gene mutation results by renal urate transporters.

Gene	Transporter	Function	Location	Gene Mutation Result
*SLC22A12*	URAT1	Reabsorbs glomerular-filtrated UA	Luminal membrane of proximal renal tubule	Hypouricemia
*SLC2A9*	GLUT9	Allows intracellular UA to exit through the basolateral side of the cells	Basolateral membrane of proximal renal tubule	Hypouricemia
*SLC17A3*	NPT4	UA excretion	Luminal membrane of proximal renal tubule	Hyperuricemia
*ABCG2*	ABCG2	UA excretion	Luminal membrane of proximal renal tubule	Hyperuricemia
*SLC17A1*	NPT1	UA excretion	Luminal membrane of proximal renal tubule	Hyperuricemia

ABCG2 = ATP-binding cassette transporter subfamily G member 2; GLUT9 = glucose transporter 9; NPT1 = sodium-dependent phosphate transporter 1; NPT4 = sodium-dependent phosphate transporter 4; UA = uric acid; URAT1 = urate transporter 1.

## Data Availability

Not applicable.
